# Modulation of Immunoregulatory Properties of Mesenchymal Stromal Cells by Toll-Like Receptors: Potential Applications on GVHD

**DOI:** 10.1155/2016/9434250

**Published:** 2016-09-21

**Authors:** Bruno Sangiorgi, Rodrigo Alexandre Panepucci

**Affiliations:** ^1^Genetics Department, Ribeirão Preto Medical School, University of São Paulo, São Paulo, SP, Brazil; ^2^Center for Cell-Based Therapy (CTC), Hemotherapy Center of Ribeirão Preto, São Paulo, SP, Brazil

## Abstract

In the last decade, the immunomodulatory properties of mesenchymal stromal cells (MSCs) have attracted a lot of attention, due to their potential applicability in the treatment of graft-versus-host disease (GVHD), a condition frequently associated with opportunistic infections. The present review addresses how Pathogen-Associated Molecular Patterns (PAMPS) modulate the immunosuppressive phenotype of human MSCs by signaling through Toll-like receptors (TLRs). Overall, we observed that regardless of the source tissue, human MSCs express TLR2, TLR3, TLR4, and TLR9. Stimulation of distinct TLRs on MSCs elicits distinct inflammatory signaling pathways, differentially influencing the expression of inflammatory factors and the ability of MSCs to suppress the proliferation of immune system cells. The capacity to enhance the immunosuppressive phenotype of MSCs through TLRs stimulation might be properly elucidated in order to improve the MSC-based immunotherapy against GVHD.

## 1. Introduction

Mesenchymal stromal cells (MSCs) are a group of adherent and fibroblastoid cells, capable of self-renewing and differentiating into osteocytes, adipocytes, and chondrocytes [1–5]. Although initially described in the bone marrow, these cells can be found virtually in all body tissues besides the perivascular niche, where they are believed to play diverse roles in tissue homeostasis [6]. In the last decade, it has been observed that, in addition to the multilineage differentiation potential, these cells also show a broad immunosuppressive potential, which extends to cells from the innate and adaptive immune system [7]. Such immunomodulatory properties raised questions about their roles in immune homeostasis and attracted attention to the potential use of MSCs in cell-based immunotherapies.

Supported by their immunosuppressive potential, several clinical studies have been conducted in order to evaluate the ability of MSCs to mitigate various disorders of the immune system. The potential of MSC-based immunotherapies has been studied in autoimmune diseases such as systemic lupus erythematosus (SLE) [8] and Crohn's disease [9, 10]; however, graft-versus-host disease (GVHD) has been the most studied so far [11]. In spite of encouraging* in vitro *studies, the effectiveness of MSC-based therapies in the treatment of GVHD has been rather variable over the clinical studies [12], revealing the need for studies regarding the factors that might account for the heterogeneous responses observed.

The variability and unsuccessfulness of MSC-based immunotherapies might be related to physical and immunological barriers. Overall, as observed by studies conducted on murine models, the trapping of infused MSCs on the lung microvasculature, possibly due to the cell size and adhesion to the vascular endothelium, might restrain the distribution of MSCs throughout the vascular system [13, 14]. However, there are observations about the immunosuppressive effect of MSCs on the alveolar microenvironment, from the paracrine signaling mechanism that might be capable of inducing the polarization of macrophages into an immunosuppressive (or M2) phenotype [15, 16]. Nevertheless, there is a possibility that the costimulatory peptides, expressed by* in vitro* expanded MSCs, might account for the depletion of the great majority of infused cells observed by studies conducted in murine models [17, 18]. On this subject, Lu and coworkers observed that the fragments of lysed MSCs might excerpt immunosuppressive properties, once the phagocytosed cell fragments are capable of inducing the acquisition of M2 phenotype by macrophages [19].

Nowadays, it is well accepted that inflammatory factors play an important role in the modulation of MSCs properties; thus, the actual inflammatory/immune state of the diseased patient may potentially greatly impact the outcome of MSC-based therapies [7, 20, 21]. A much less appreciated aspect in this context is the impact that pathogenic infections, commonly associated with the patient's condition [22, 23], may have in the outcome of MSC-based therapies. During infections, Pathogen-Associated Molecular Patterns (PAMPs) are recognized by Toll-like receptors (TLRs), present in diverse cells of the innate immune system, modulating their responses [24–26]. The objective of this review is to provide a broad overview of how MSCs derived from human tissues may be modulated by the interaction of PAMPs and TLRs, as well as possible therapeutic implications and future directions.

## 2. Graft-versus-Host Disease

Graft-versus-host disease is a potentially fatal disease that occurs in patients undergoing transplantation of hematopoietic stem cells, triggered by immunological incompatibilities between T cells derived from donor and host's antigen-presenting cells, resulting in an exacerbated inflammatory response and damage to various organs and tissues, especially the skin, liver, and gastrointestinal (GI) tract [22]. The GI damage poses a threat to the integrity of intestine's innate defense epithelial barrier, comprised by the mucosal layer, innate antimicrobial peptides and innate lymphoid cells that act together in the retention and tolerance of commensal bacteria that inhabit the intestinal lumen [27]. The damage of the intestinal barrier promotes translocation of intestinal bacteria fragments, as observed by the presence of circulating lipopolysaccharide (LPS) in experimental studies of bone marrow transplantation conducted on murine models [28]. The amplification of the inflammatory response due to translocating PAMPs appears to be crucial to the increase in the severity of GVHD, considering that studies in murine models [29, 30] and also clinical studies [31–33] have shown a reduction in the incidence and severity of the disease, when study subjects were submitted to intestinal decontamination prior to the transplant.

Because of the pathophysiology of GVHD and the treatment used to combat the disease, transplanted patients are characteristically immunosuppressed, with reduced quantities of distinct sets of innate immune system cells, what in turn makes the patients highly susceptible to infections [34]. Patients can be affected both by viral infections stemming from the reactivation of latent viruses, such as cytomegalovirus and herpes simplex virus, or through new infections following transplantation, such as the influenza virus [35]. Although control measures may successfully mitigate the reactivation of opportunistic viruses, diseases caused by fungi, especially* Aspergillus*, are the major cause of infectious mortality and morbidity after transplantation [36, 37]. In addition, immunocompromised patients have a prolonged risk of infection by bacteria, like* Streptococcus pneumoniae*,* C. difficile colitis*,* Mycobacterium sp.* and antibiotic-resistant bacteria [35].

## 3. Toll-Like Receptors

Cells of the innate immune system distinguish the body's own molecules (self) from those belonging to the pathogen (non-self) through specific receptors, such as Toll-like receptors (TLR), a group of 10 functional proteins in humans [38]. Toll-like receptors are type I transmembrane proteins with an N-terminal domain-containing leucine-rich repeats, which are responsible for recognition of ligands, as well as a C-terminal region containing a Toll/interleukin-1 (TIR) domain. In cells of the innate immune system, TLRs are present as homodimers (formed by the same receptor) or heterodimers (consisting of different receptors) that recognize PAMPs derived from bacteria, fungi, and viruses [39]. Importantly, the location of the Toll-like receptors is associated with the nature of the ligand recognized (Table 1), once the protein and lipid receptors (such as TLR2 and TLR4) are distributed in the surface plasma membrane, while nucleic acid receptors (such as TLR3 and TLR9) are located in intracellular compartments such as endoplasmic reticulum, endosomes and lysosomes [40].

Studies on the functions of TLRs performed on antigen-presenting cells (APC) have shown that the stimulation of these receptors has the effect of inducing the expression of lymphocyte costimulatory proteins, as well as the secretion of inflammatory cytokines that are crucial for the recruitment and activation of lymphocytes in order to combat infection by pathogens (Table 1) [25]. Toll-like receptors can activate the nuclear factor kappa B (NF-*κ*B) pathway through two distinct signaling cascades: the myeloid differentiation primary response 88- (MyD88-) dependent and Toll/IL-1 receptor domain-containing adaptor inducing IFN-beta (TRIF-) dependent (also known as “MyD88-independent”) pathways. With the exception of TLR3, the stimulation of all Toll-like receptors leads to the activation of MyD88, responsible for binding to TLR, as well as a death domain (DD) responsible for its connection to IL-1 receptor-associated kinases (IRAKs) [41]. IRAKs activation leads to the recruitment and activation of tumor necrosis factor receptor-associated factor-6 (TRAF-6), which assists in the activation of the I*κ*B kinase (IKK) complex and subsequent degradation of inhibitor of kappa B (IkB), resulting in the release and nuclear translocation of NF-*κ*B transcription factor, leading to the expression of proinflammatory cytokines [42]. In plasmacytoid dendritic cells (pDCs), signaling through the MyD88-dependent pathway (downstream TLR7 or TLR9) activates NF-*κ*B and IFN-regulatory factor-7 (IRF-7) with the consequent expression of type I IFN genes, mediated by an association between IRF-7 and TRAF-6 [43]. The activation of TRIF-dependent pathway occurs after the stimulation of TLR3 or TLR4, when the latter is in endosomal compartments of phagosomes resulting in the activation of NF-*κ*B, as well as IFN-regulatory factor-3 (IRF-3) and IRF-7 [44, 45]. Table 1 shows the different PAMPs and the cognate TLRs associated with their recognition (and their localization in the cell), as well as the main downstream effects.

Although TLRs stimulation, in general, induces the expression of proinflammatory cytokines, TLR9 stimulation may induce an anti-inflammatory phenotype on dendritic cells. As suggested by Puccetti and collaborators, the mechanism underlying the acquisition of an anti-inflammatory phenotype of TLR9-stimulated DCs might result from the expression of type I interferons, which bind to interferon receptors in an autocrine fashion, leading to the activation of the anti-inflammatory noncanonical NF-*κ*B pathway and consequently IDO synthesis, which induces differentiation of regulatory T cells [46]. Overall, depending on the nature of the receptor, ligand, and cell type, distinct inflammatory pathways may be activated, leading to different effects on a given cell of the innate immune system.

## 4. The Modulatory Activity of Inflammatory Factors on the Immunoregulation Mediated by Mesenchymal Stromal Cells

So far, several* in vitro* studies have observed the ability of MSCs from bone marrow (BM), adipose tissue (AT), and umbilical cord (UC) to suppress or modulate the immunological properties of various cells of the innate and adaptive immune system [47]. During this event of immunosuppression, MSCs act through paracrine signaling and cell-cell contacts. Interestingly, studies to date have observed that the presence of an inflammatory environment has the ability to drastically alter the immunosuppressive potential of MSC, through changes in the expression of both adhesion molecules and soluble factors [48, 49]. However, studies conducted on human and murine MSCs observed a similar immune suppressive capacity on cocultures with direct contact or separated by a membrane permeable to soluble factors (transwell), what indicates a major role of paracrine signaling mechanism over cell contact on immune suppression [50, 51], particularly, by secreted soluble factors such as transforming growth factor beta (TGF-*β*), hepatocyte growth factor (HGF), prostaglandin E2 (PGE-2), interleukin-10 (IL-10), and IDO [20, 52–54].

The inflammatory environment is capable of modulating the expression of pro- and anti-inflammatory factors produced by MSCs from different sources. In a study conducted by Prasanna and coworkers, it was noticed that stimulation by IFN-*γ* was able to increase IDO gene expression and to enhance the immunosuppressive activity of MSCs derived from both bone marrow and Wharton's jelly. On the other hand, only the latter showed an increase in transcript and protein levels of HGF after TNF-*α* stimulation, indicating that the responsiveness of MSCs to inflammatory factors may vary depending on its source [48]. The modulation of the immunosuppressive capacity was also observed by DelaRosa and coworkers, as IFN-*γ* treatment, but not TNF-*α*, resulted in an increase in the immunosuppressive capacity mediated by soluble factors, on MSCs derived from adipose tissue [55]. In contrast, English and coworkers found no changes in the immunosuppressive function of murine BM- MSCs after stimulation with IFN-*γ* or TNF-*α*, although both cytokines regulated, at different levels, the expression of anti-inflammatory factors [56].

Altogether, previous studies showed that IFN-*γ* stimulation may enhance the immunosuppressive properties of MSCs, what corroborates the hypothesis that mesenchymal stromal cells need to be licensed in order to reach their maximum immunosuppressive potential [7]. Importantly, inflammatory cytokines, such as IFN-*γ* and TNF-*α*, activate signaling pathways common to those downstream of Toll-like receptors, upon recognition of PAMPs [57]. Thus, the study of PAMP-TLR signaling is of critical importance to gain knowledge on the factors that might modulate the immunosuppressive properties exerted by mesenchymal stromal cells.

## 5. The Expression of Toll-Like Receptors on Mesenchymal Stromal Cells

To date, several* in vitro* studies have been conducted in order to assess the presence and function of Toll-like receptors, especially TLR2, TLR3, TLR4, and TLR9, in human MSCs obtained from different sources such as bone marrow, adipose tissue, and umbilical cord. The expression of Toll-like receptors in MSC, from different sources, has been evaluated in several studies at the transcript or protein level (Table 2). So far, bone marrow was the most common MSCs source in the studies evaluating the presence of TLR, while fewer studies used cells derived from adipose tissue and umbilical cord. In general, transcripts of Toll-like receptors: TLR2, TLR3, TLR4, and TLR9 were reported to be expressed in MSCs from all sources [58–62]. However, there are reports claiming the absence of TLR4 transcripts on UC-MSC [61] and TLR9 on BM-MSCs [63, 64]. Interestingly, all studies observed the expression of transcripts for TLR2, TLR3, TLR4, and TLR9, in MSCs derived from adipose tissue [58, 60, 61, 65].

At the protein level, independent studies observed that cells derived from bone marrow and adipose tissue express TLR2, TLR3, TLR4, and TLR9 (Table 2) [60, 62, 66]. However, the expression of these receptors on UC-MSCs is still incompletely appreciated, as Chen and coworkers found no expression of TLR2 and TLR9 [59], while TLR4 was not identified by Raicevic and coworkers [61]. Importantly, independent studies show that, in general, transcript and protein expression are correlated [60–65].

Based on these results, it is clear that the expression profile of TLRs in MSCs is not similar to that presented by pDCs (which only express TLR7 and TLR9), resembling more macrophages, which express virtually all Toll-like receptors, although the expression of TLR7 and TLR9 in MSCs occurs at lower levels, as compared to pDCs [67]. Therefore, MSCs are equipped with the TLRs machinery to activate both the inflammatory NF-*κ*B pathway and interferon regulatory factors. These findings may have potential applications on immunotherapies, what requires a deeper investigation, through functional studies, in order to identify the molecular pathways activated after the stimulation of Toll-like receptors in human mesenchymal stromal cells.

## 6. The Modulatory Effect Exerted by Toll-Like Receptors on the Expression of Inflammatory Factors by Mesenchymal Stromal Cells

To date, studies conducted on human MSCs observed that mesenchymal stromal cells, derived from various sources, have a functional signaling cascade of Toll-like receptors. Similar to what is observed on innate immune system cells, the study conducted by Chen and coworkers in UC-MSCs described that the stimulation of Toll-like receptors TLR2 and TLR4, but not TLR3 and TLR9, leads to the activation of the NF-*κ*B pathway [59]. Moreover, studies performed with BM-MSCs found the presence of NF-*κ*B in the nuclear extracts after the stimulation with LPS and polyinosinic-polycytidylic acid (POLY-IC) [64] and a synthetic dsRNA, as well as the phosphorylation of IKK*α*/*β* [62]. Ik*β*-*α* degradation was observed by Lombardo and coworkers, on AT-MSC, after the stimulation of TLR2, TLR3, or TLR4 [65].

As expected for the activation of inflammatory pathways, the stimulation of Toll-like receptors TLR2, TLR3, TLR4, and TLR9, in general, is followed by increased expression of proinflammatory cytokines (Table 3), mainly interleukin-6 (IL-6) and interleukin-8 (IL-8), regardless of the source of MSC [59, 60, 62, 64, 65, 70]. Studies conducted on AT-MSCs have also found an increased expression of IL-1*β* [60, 61] and TNF-*α* [60] following TLRs stimulation, which was found to be unaltered by Lombardo and coworkers [65]. Interestingly, while the study conducted by Raicevic and coworkers has found a similar level of increase in the protein expression of IL-6 and IL-8 after the stimulation of LPS or POLY-IC [61], Lombardo and coworkers have found a greater increase in the levels of those cytokines after the stimulation with POLY-IC, in comparison to PGN and LPS, which also led to a more rapid degradation of Ik*β*-*α*, a repressor of NF-*κ*B [65].

Studies conducted on BM-MSC, in general, have found that the stimulation with LPS or POLY-IC led to an increase in the expression of proinflammatory cytokines IL-1*β*, IL-6, and IL-8 [61–63, 68, 70]. However, despite contrasting findings, independent studies have observed a more pronounced increase of IL-6 and IL-8 expression after the stimulation with LPS in comparison to POLY-IC [68, 70, 77]. The effects of TLR3 or TLR4 stimulation regarding alterations on TNF-*α* expression levels are less clear, as independent studies have observed a maintenance [68, 75] or even reduction [62, 78] of its expression level. Likewise, there is a current need for further studies on TLR9 stimulation on BM-MSC, once our findings indicate that TLR9 stimulation maintained the expression of proinflammatory factors (with exception of TNF-*α* that was reduced) [66], which were found to increase by Tomchuck and coworkers [62].

Similar to what was found on different sources of mesenchymal stromal cells, in general, studies conducted on UC-MSCs have observed an increase in the expression levels of IL-6 and IL-8 following stimulation with LPS or POLY-IC [59, 71, 72]. Interestingly, Fuenzalida and coworkers observed that, in comparison to TLR3 stimulation, TLR4 stimulation led to higher increases in IL-6 and IL-8 expression levels [72]. This differential modulation in IL-8 expression levels upon stimulus with LPS or POLY-IC was similarly observed by Chen and coworkers and Zhao and coworkers, while IL-6 levels increased similarly, regardless of the stimulus used [59, 71]. Taken together, the findings of independent studies conducted on MSCs from different sources indicate that although the stimulation of TLR3 and TLR4 leads to the activation of the NF-*κ*B pathway, the upregulation of proinflammatory cytokines is more pronounced on TLR4-stimulated MSC. In addition to inducing changes in the profile of proinflammatory factors secreted by MSCs, the stimulation of Toll-like receptors can also modulate the expression of anti-inflammatory factors, mainly IDO and TGF-*β* [61, 64–66, 77]. Up to date, few studies have been conducted on AT-MSCs that have observed that TLRs stimulation did not alter the expression of anti-inflammatory factors [58, 61, 65]. However, Lombardo and coworkers observed that the stimulation with POLY-IC, but not PGN or LPS, was followed by an increase in IDO activity in AT-MSCs, but only in the highest concentration evaluated (10 *μ*g/mL) [65]. In line with these results, Mancheño-Corvo and coworkers observed that TLR3 stimulation on AT-MSCs also increased IDO activity, as well as PGE2 protein levels [73].

Similar to AT-MSCs, due to the contrasting findings so far, the effects of POLY-IC in the induction of an anti-inflammatory phenotype on BM-MSCs are still unclear. Overall, the studies conducted up to now indicate that the stimulation of TLR3 leads to an increase in the expression or activity of IDO [76, 77], as well as a reduction in the expression of TGF-*β* [61, 70, 75, 77]. Likewise, independent studies observed that TLR4 stimulation on BM-MSCs was followed by an increase in the levels or activity of IDO [76, 77] and maintenance [64, 70] or reduction [61, 77] of TGF-*β* levels. So far, the effects of TLR9 stimulation on the expression of anti-inflammatory factors on BM-MSCs were not properly described; nevertheless, we observed that treatment with the CpG oligodeoxynucleotide (CpG-ODN) DSP30 led to slight increase on the gene expression of TGF-*β*. To date, there were few studies about the capacity of TLRs stimulation to modulate the expression of anti-inflammatory factors on UC-MSCs; however, the findings indicate that the stimulation of TLR2, TLR3, or TLR9 leads to an upregulation of IDO [59, 71, 72, 79].

Taken together, several independent studies noted an increased expression and/or activity of IDO after the stimulation of Toll-like receptors in cells derived from umbilical cord [59, 71], bone marrow [76, 77], and adipose tissue [65]. Strikingly, Opitz and coworkers observed that IDO expression following TLR3 or TLR4 engagement on BM-MSCs is dependent on an autocrine loop, initiated by the activation of the TRIF-dependent pathway and expression of IFN-*β*, which interacts with IFNAR and elicits the phosphorylation of STAT-1, leading to the expression of IDO [76]. In line with this observation, Chen and coworkers observed the phosphorylation of IRF-3 following TLR3 and TLR9 stimulation on UC-MSCs, as well as an increase in the expression of IFN-*β* and IDO [59].

## 7. The Modulatory Effect Exerted by Toll-Like Receptors on the Suppressive Capacity of Mesenchymal Stromal Cells

In order to evaluate changes in the ability of MSCs to suppress the proliferation of lymphocytes, due to the stimulation with toll-like receptor, in general, immunosuppression assays were performed using mixed lymphocyte reaction (MLR) [61, 63, 64, 76, 77]. Proliferation of the lymphocytes was measured by incorporation of 5-bromo-2′-deoxyuridine (BrDU) during DNA synthesis [59, 61, 63], or the fluorescence loss of the intracellular dye carboxyfluorescein succinimidyl ester (CFSE) [65, 73, 77] during cell divisions. Importantly, results measured by BrDU incorporation and CFSE loss are comparable [55, 80].

Studies of Opitz and coworkers, as well as Waterman and coworkers, observed an increase in lymphocytes suppression mediated by BM-MSCs after the stimulation of TLR3 [76, 77]; however, the opposite was observed in the studies of Raicevic and coworkers and also Liotta and coworkers [63, 64]. In accordance with the increased expression of proinflammatory cytokines, observed in aforementioned studies, a greater number of studies found a reduction in the suppressive potential of BM-MSCs, after TLR4 stimulation [63, 64, 66, 77]. Despite the current need for further studies to elucidate the effects driven by TLR9 stimulation on the suppressive capacity of BM-MSCs, we have shown an increase on the suppressive capacity after the treatment with CpG-ODN in a MSCs/T-lymphocytes coculture setup [66].

To date, a very limited number of studies have been carried out to evaluate the suppressive potential of MSCs derived from umbilical cord and adipose tissue, following the stimulation with Toll-like receptors. In the study by Chen and coworkers, it was observed that the stimulation of TLR3 or TLR9 on UC-MSCs increased the suppression of lymphocyte proliferation, what would be related to the induction of IDO expression, which was unaltered after stimulation of TLR2 and TLR4 [59]. Likewise, Fuenzalida and coworkers observed an increase and maintenance on the suppressive potential of UC-MSCs following the stimulation of TLR3 and TLR4, respectively, which also might be related to the induction of IDO expression [72]. Interestingly, despite the increased expression of proinflammatory cytokines following the stimulation of Toll-like receptors, observed in several studies on AT-MSCs, studies conducted by Raicevic and Lombardo showed that the suppressive potential was unchanged after stimulation with various TLRs ligands [61, 65].

## 8. Concluding Remarks

Up to date, several clinical trials were performed using BM-MSCs to ameliorate GVHD, with variable results [12]. Besides physical and immunological barriers, the variability of MSC-based immunotherapies might be related to the modulatory activity driven by TLRs stimulation on the immune phenotype of MSCs. In this review, we addressed the capacity of TLRs stimulation to modulate the expression of pro- and anti-inflammatory factors, as well as the suppressive activity exerted by MSCs on activated immune system cells.

In order to provide a broad overview of the studies evaluating the roles of TLR signaling in MSCs, our review had to limit the specific discussion of potential factors that might account for the heterogeneity of the results presented by different studies conducted so far. For instance, differences in concentration, treatment time, and molecular structure of the TLRs agonists, which may impact substantially the results observed, were not addressed by us. Despite the specificity of lipopolysaccharide towards TLR4, contamination with lipopeptides may occur depending on the method of extraction of this bacterial component, leading to an unwanted stimulation of TLR2 [81]. Differences in the concentration and molecular weight of the TLR3 ligand POLY-IC may result in different levels of IRF-3 activation and type I interferon expression [82]. The CpG oligodeoxynucleotides (CpG-ODNs) synthetic constructs may be grouped into three classes (A, B, and C) according to differences in molecular structure and the effect on immune system cells, especially B lymphocytes and dendritic cells [83–87]; thus, different constructs used may also differentially impact the immunosuppressive properties of MSC.

As a whole, independent studies have observed the gene and protein expression of Toll-like receptors: TLR2, TLR3, TLR4, and TLR9 in human mesenchymal stromal cells from different sources [58–62]. Additionally, it was observed that the stimulation of those receptors on MSCs might elicit the activation of signaling pathways also observed on innate immune system cells, as NF-*κ*B [62, 65] and IRF [59] transcription factors. Altogether, those observations indicate that MSCs have a functional signaling cascade of Toll-like receptors, which is especially relevant considering the potential use of MSCs on immunotherapies against GVHD, a disease often affected by associated infections [22, 23].

The activation of NF-*κ*B in MSCs, by peptidoglycan, LPS, dsRNA, or CpG-ODN, has worrying implications for cell-therapy, given that such ligands mimic PAMPs found in several pathogens that commonly affect GVHD patients. Depending on the pathogens and PAMPs milieu present in the patient that receives the MSCs infusion, the stimulation of TLRs on infused MSCs might hamper their immunosuppressive properties or even enhance the inflammatory process.

Besides the activation of the NF-*κ*B pathway, the stimulation of TLR3, TLR4, and TLR9 was able to activate the transcription factor IRF in mesenchymal stromal cells, leading to IFN-*β* expression, which might have led to the activation of IDO synthesis by an autocrine signaling mechanism. The synthesis of IFN-*β* after the stimulation of Toll-like receptors has the potential to enhance the effectiveness of MSC-based immunotherapy, in view of the ability of this cytokine to reduce the capacity of dendritic cells to present antigens [88], a crucial event on the initiation of GVHD [89]. Stimulation with IFN-*β* inhibits the differentiation of Th17 cells, a group of cells with an active role in autoimmune diseases [90], besides inducing the differentiation of Treg cells [91]. Treatment with IFN-*β* has been effective in promoting the survival of patients with multiple sclerosis, an autoimmune neurodegenerative disease, and it has not been associated with life-threatening adverse effects during long-term treatment [92, 93]. Thus, specific TLRs ligands could be used for the* ex vivo* priming of MSC, aiming to increase their immunosuppressive properties.

This study addressed the alterations of the cytokines that were most commonly evaluated on mesenchymal stromal cells; therefore, there is a need to study the effects of TLR signaling on the expression of additional anti-inflammatory or proinflammatory factors. For instance, our group recently observed that the stimulation of TLR9 on BM-MSCs led to a significant increase on the expression of ectonucleotidases involved on the synthesis of adenosine, a potent immunosuppressant, as well as a slight increase in adenosine synthesis [66]. Amarnath and coworkers demonstrated, in a xenogeneic human-into-mouse GVHD model, that MSCs infusion led to an increased amount of exosomes competent to produce adenosine and, moreover, the immunosuppression mediated by MSCs was mitigated after the addition of adenosine receptor inhibitors [94].

As a whole, the results obtained by different* in vitro* studies suggest that the stimulation of Toll-like receptors on human mesenchymal stromal cells could have beneficial or detrimental effects in the immunotherapy against GVHD. Additional mechanisms related to the regulatory effect of TLRs stimulation on the survival of infused MSCs, an important barrier to the success of the MSC-based immunotherapies, might also prove relevant. On this subject, Giuliani and coworkers observed that TLR3 triggering, but not TLR4, protects BM-MSCs from lysis by NK cells, which may have been influenced by the dissociation of surface MICA on BM-MSCs [70]. Overall, a clear understanding of TLRs signaling in MSCs has the potential to improve the manipulation of these cells in order to direct a specific anti-inflammatory behavior.

## Figures and Tables

**Table 1:** Recognition and signaling by Toll-like receptors.

PAMP	TLR	Transcription factors	Target genes
Triacyl lipopeptide	TLR1/2^*∗*^	NF-*κ*B	Proinflammatory cytokines
Diacyl lipopeptide	TLR2/6^*∗*^	NF-*κ*B	Proinflammatory cytokines
dsRNA	TLR3^#^	IRF	Type I INF
LPS	TLR4^*∗*^	NF-*κ*B and IRF	Proinflammatory cytokines and type I INF
Flagellin	TLR5^*∗*^	NF-*κ*B	Proinflammatory cytokines
ssRNA	TLR7/8^#^	NF-*κ*B and IRF (pDCs)	Proinflammatory cytokines and type I INF (pDCs)
CpG-DNA	TLR9^#^	NF-*κ*B and IRF (pDCs)	Proinflammatory cytokines and type I INF (pDCs)

*Notes*. PAMPs derived from viruses, bacteria, and fungi are recognized by Toll-like receptors, in homodimers or heterodimers, resulting in the activation of transcription factors with subsequent transcription of target genes. ^*∗*^Located in the plasma membrane. ^#^Located in intracellular compartments.

**Table 2:** Gene and protein expression of Toll-like receptors MSCs from different sources.

Source	Gene expression				Protein expression			
	TLR2	TLR3	TLR4	TLR9	TLR2	TLR3	TLR4	TLR9
AT	P [58, 60, 61, 65]	P [50, 58, 60, 61, 65]	P [50, 58, 60, 61, 65]	P [58, 60, 61, 65]	P [60, 65]	P [60, 61]	P [60, 61, 65]	P [60]
BM	P [61–64, 68]	P [50, 61–64, 68, 69]	P [50, 61–64, 68, 69]	A [63, 64] P [61, 62, 68]	P [62, 63, 66]	P [61–64, 66, 68, 70]	P [61–64, 66, 70]	P [62, 66]
UC	P [59, 61, 71]	P [50, 59, 61, 71, 72]	A [61] P [50, 59, 71, 72]	P [59, 61, 71]	A [59] P	P [59, 61]	A [61] P [59]	A [59]

*Notes*. presence or absence of Toll-like receptors: TLR2, TLR3, TLR4, and TLR9, evaluated at gene or protein expression levels on mesenchymal stromal cells from different sources. Gene expression was evaluated by techniques of quantitative PCR or reverse-transcription PCR, while protein expression was evaluated by western blot or flow cytometry. AT (adipose tissue), BM (bone Marrow), and UC (umbilical cord). P: presence; A: absence.

**Table 3:** Effects of Toll-like receptors on MSCs from different sources.

Source	PAMP	Proinflammatory factors				Anti-inflammatory factors			Suppressive potential
		IL-1*β*	IL-6	IL-8	TNF-*α*	IDO^*∗*^	PGE2	TGF-*β*	
AT	PGN	↑[60] =[65]	↑[65]	↑[65] =[60]	↑[60] =[65]	=[65]			=[65]
	POLY-IC	↑[58, 60, 61] =[65]	↑[58, 61, 65]	↑[58, 61, 65] =[60]	↑[58, 60] =[65]	↑[65, 73]	↑[73] =[61]	=[58] ↓[61]	↑[73] =[61, 65]
	LPS	↑[60, 61] =[65]	↑[61, 65]	↑[61, 65] =[60]	↑[60] =[65]	=[65]	=[61]	↓[61]	=[61, 65]
	CpG-ODN	↑[60]	↑[65]	=[60]	=[60]				

BM	PGN	↑[74] =[63]	↑[63]	↑[74]				↑[63]	
	POLY-IC	↑[61–63, 68, 70] =[64, 68]	↑[61–64, 68–70, 75, 76] =[77]	↑[61, 62, 64, 68–70, 75, 77]	=[68, 75] ↓[62]	↑[76, 77] =[64]	↑[70, 77] =[61, 78]	↑[63] =[64] ↓[61, 70, 75, 77]	↑[76, 77] =[61] ↓[63, 64]
	LPS	↑[61, 63, 66, 68, 70, 74] =[62, 64]	↑[61–64, 66, 68–70, 75–77] =	↑[61, 62, 64, 66, 68, 70, 74, 75, 77] =	↑ =[66, 68, 75] ↓[62, 78]	↑[76, 77] =[64]	↑[70, 78] =[61, 77]	↑[63] =[64, 66, 70] ↓[61, 77]	↑[76] =[61] ↓[63, 64, 66, 77]
	CpG-ODN	↑[62] =[66]	↑[62] =[66]	↑[62, 66]	↑[62] ↓[66]			↑[66]	↑[66]

UC	PGN								=[59]
	POLY-IC	=[61]	↑[59, 71, 72] =[61]	↑[59, 71, 72] =[61]		↑[59, 71, 72]		=[61] ↓[71]	↑[59, 72] =[61]
	LPS	↑[79] =[61]	↑[59, 71, 72, 79] =[61]	↑[59, 71, 72, 79] =[61]		↑[71, 79] =[72]		=[61] ↓[71]	=[59, 61, 72]
	CpG-ODN		=[59] ↓[71]	=[59] ↓[71]		↑[59] ↓[71]		↓[71]	↑[59]

*Notes*. Observations of the effects driven by the stimulation of different TLRs, on mesenchymal stromal cells from different sources, on the gene and protein expression of proinflammatory cytokines: IL-1*β*, IL-6, IL-8, and TNF-*α*, gene and protein expression of anti-inflammatory factors: IDO1 (IDO), TGF-*β*1 (TGF-*β*), and PGE2, and the capacity to suppress the proliferation of lymphocytes (suppressive potential). ↑: increase; =: no alteration; ↓: reduction. ^*∗*^Alterations of IDO expression levels or activity to induce tryptophan catabolism.

## Abbreviations

AD: Adipose tissue
BM: Bone marrow
BrDU: 5-Bromo-2′-deoxyuridine
CD: Cluster of differentiation
CFSE: Carboxyfluorescein succinimidyl ester
CpG-ODN: CpG oligodeoxynucleotide
HGF: Hepatocyte growth factor
ICAM-1: Intercellular Adhesion Molecule 1
IFN-y: Interferon gamma
I*κ*B: Inhibitor of kappa B
IKK: I*κ*B kinase
IL: Interleukin
IRAK: IL-1 receptor-associated kinase
IRF: IFN-regulatory factor
LPS: Lipopolysaccharide
MLR: Mixed lymphocyte reaction
MSCs: Mesenchymal stromal cells
MyD88: Myeloid differentiation primary response 88
NF-*κ*B: Nuclear factor kappa B
PGE-2: Prostaglandin E2
POLY-IC: Polyinosinic-polycytidylic acid
TGF-*β*: Transforming growth factor beta
TLR: Toll-like receptor
TNF-*α*: Tumor necrosis factor alpha
TRAF: Tumor necrosis factor receptor-associated factor
TRIF: Toll/IL-1 receptor domain-containing adaptor inducing IFN-beta
UC: Umbilical cord
VCAM-1: Vascular cell adhesion molecule 1.

## References

[1] Friedenstein A. J., Piatetzky S., Petrakova K. V. (1966). Osteogenesis in transplants of bone marrow cells. *Journal of Embryology and Experimental Morphology*.

[2] Afanasyev B. V., Elstner E. E., Zander A. R. (2009). A. J. Friedenstein, founder of the mesenchymal stem cell concept. *Cellular Therapy and Transplantation*.

[3] Wong R. S. (2011). Mesenchymal stem cells: angels or demons?. *Journal of Biomedicine and Biotechnology*.

[4] Tavassoli M., Crosby W. H. (1968). Transplantation of marrow to extramedullary sites. *Science*.

[5] Caplan A. I. (1991). Mesenchymal stem cells. *Journal of Orthopaedic Research*.

[6] Covas D. T., Panepucci R. A., Fontes A. M. (2008). Multipotent mesenchymal stromal cells obtained from diverse human tissues share functional properties and gene-expression profile with CD146^+^ perivascular cells and fibroblasts. *Experimental Hematology*.

[7] English K. (2013). Mechanisms of mesenchymal stromal cell immunomodulation. *Immunology and Cell Biology*.

[8] Carrion F. A., Figueroa F. E. (2011). Mesenchymal stem cells for the treatment of systemic lupus erythematosus: is the cure for connective tissue diseases within connective tissue?. *Stem Cell Research and Therapy*.

[9] Duijvestein M., Vos A. C. W., Roelofs H. (2010). Autologous bone marrow-derived mesenchymal stromal cell treatment for refractory luminal Crohn's disease: results of a phase I study. *Gut*.

[10] Ciccocioppo R., Bernardo M. E., Sgarella A. (2011). Autologous bone marrow-derived mesenchymal stromal cells in the treatment of fistulising Crohn's disease. *Gut*.

[11] Wang S., Qu X., Zhao R. C. (2012). Clinical applications of mesenchymal stem cells. *Journal of Hematology & Oncology*.

[12] Kim N., Im K., Lim J. (2013). Mesenchymal stem cells for the treatment and prevention of graft-versus-host disease: experiments and practice. *Annals of Hematology*.

[13] Schrepfer S., Deuse T., Reichenspurner H., Fischbein M. P., Robbins R. C., Pelletier M. P. (2007). Stem cell transplantation: the lung barrier. *Transplantation Proceedings*.

[14] Fischer U. M., Harting M. T., Jimenez F. (2009). Pulmonary passage is a major obstacle for intravenous stem cell delivery: the pulmonary first-pass effect. *Stem Cells and Development*.

[15] Lee J. W., Fang X., Krasnodembskaya A., Howard J. P., Matthay M. A. (2011). Concise review: mesenchymal stem cells for acute lung injury: role of paracrine soluble factors. *STEM CELLS*.

[16] Ionescu L., Byrne R. N., van Haaften T. (2012). Stem cell conditioned medium improves acute lung injury in mice: in vivo evidence for stem cell paracrine action. *American Journal of Physiology—Lung Cellular and Molecular Physiology*.

[17] Crop M. J., Korevaar S. S., de Kuiper R. (2011). Human mesenchymal stem cells are susceptible to lysis by CD8(+) T cells and NK cells. *Cell Transplantation*.

[18] Eggenhofer E., Benseler V., Kroemer A. (2012). Mesenchymal stem cells are short-lived and do not migrate beyond the lungs after intravenous infusion. *Frontiers in Immunology*.

[19] Lu W., Fu C., Song L. (2013). Exposure to supernatants of macrophages that phagocytized dead mesenchymal stem cells improves hypoxic cardiomyocytes survival. *International Journal of Cardiology*.

[20] Yagi H., Soto-Gutierrez A., Parekkadan B. (2010). Mesenchymal stem cells: mechanisms of immunomodulation and homing. *Cell Transplantation*.

[21] Ghannam S., Bouffi C., Djouad F., Jorgensen C., Noël D. (2010). Immunosuppression by mesenchymal stem cells: mechanisms and clinical applications. *Stem Cell Research and Therapy*.

[22] Jacobsohn D. A., Vogelsang G. B. (2007). Acute graft versus host disease. *Orphanet Journal of Rare Diseases*.

[23] Ulff-Møller C. J., Nielsen N. M., Rostgaard K., Hjalgrim H., Frisch M. (2010). Epstein-Barr virus-associated infectious mononucleosis and risk of systemic lupus erythematosus. *Rheumatology*.

[24] Volpi C., Fallarino F., Bianchi R. (2012). A GpC-rich oligonucleotide acts on plasmacytoid dendritic cells to promote immune suppression. *The Journal of Immunology*.

[25] Takeda K., Akira S. (2005). Toll-like receptors in innate immunity. *International Immunology*.

[26] Hayden M. S. (2004). Signaling to NF-*κ*B. *Genes & Development*.

[27] Brown E. M., Sadarangani M., Finlay B. B. (2013). The role of the immune system in governing host-microbe interactions in the intestine. *Nature Immunology*.

[28] Hill G. R., Ferrara J. L. M. (2000). The primacy of the gastrointestinal tract as a target organ of acute graft-versus-host disease: rationale for the use of cytokine shields in allogeneic bone marrow transplantation. *Blood*.

[29] van Bekkum D. W., Roodenburg J., Heidt P. J., van der Waaij D. (1974). Mitigation of secondary disease of allogeneic mouse radiation chimeras by modification of the intestinal microflora. *Journal of the National Cancer Institute*.

[30] Jones J. M., Wilson R., Bealmear P. M. (1971). Mortality and gross pathology of secondary disease in germfree mouse radiation chimeras. *Radiation Research*.

[31] Beelen D. W., Elmaagacli A., Müller K.-D., Hirche H., Schaefer U. W. (1999). Influence of intestinal bacterial decontamination using metronidazole and ciprofloxacin or ciprofloxacin alone on the development of acute graft-versus-host disease after marrow transplantation in patients with hematologic malignancies: final results and long-term follow-up of an open-label prospective randomized trial. *Blood*.

[32] Vossen J. M., Guiot H. F. L., Lankester A. C. (2014). Complete suppression of the gut microbiome prevents acute graft-versus-host disease following allogeneic bone marrow transplantation. *PLoS ONE*.

[33] Vossen J. M., Heidt P. J., van den Berg H., Gerritsen E. J. A., Hermans J., Dooren L. J. (1990). Prevention of infection and graft-versus-host disease by suppression of intestinal microflora in children treated with allogeneic bone marrow transplantation. *European Journal of Clinical Microbiology & Infectious Diseases*.

[34] Seggewiss R., Einsele H. (2010). Immune reconstitution after allogeneic transplantation and expanding options for immunomodulation: an update. *Blood*.

[35] Marr K. A. (2012). Delayed opportunistic infections in hematopoietic stem cell transplantation patients: a surmountable challenge. *Hematology/The Education Program of the American Society of Hematology*.

[36] Chen C.-S., Boeckh M., Seidel K. (2003). Incidence, risk factors, and mortality from pneumonia developing late after hematopoietic stem cell transplantation. *Bone Marrow Transplantation*.

[37] Marr K. A., Bowden R. A. (1999). Fungal infections in patients undergoing blood and marrow transplantation. *Transplant Infectious Disease*.

[38] Aderem A., Ulevitch R. J. (2000). Toll-like receptors in the induction of the innate immune response. *Nature*.

[39] West A. P., Koblansky A. A., Ghosh S. (2006). Recognition and signaling by toll-like receptors. *Annual Review of Cell and Developmental Biology*.

[40] Blasius A. L., Beutler B. (2010). Intracellular toll-like receptors. *Immunity*.

[41] Wesche H., Henzel W. J., Shillinglaw W., Li S., Cao Z. (1997). MyD88: an adapter that recruits IRAK to the IL-1 receptor complex. *Immunity*.

[42] Kawagoe T., Sato S., Matsushita K. (2008). Sequential control of Toll-like receptor-dependent responses by IRAK1 and IRAK2. *Nature Immunology*.

[43] Kawai T., Sato S., Ishii K. J. (2004). Interferon-*α* induction through Toll-like receptors involves a direct interaction of IRF7 with MyD88 and TRAF6. *Nature Immunology*.

[44] Gohda J., Matsumura T., Inoue J.-I. (2004). Cutting edge: TNFR-associated factor (TRAF) 6 is essential for MyD88-dependent pathway but not Toll/IL-1 receptor domain-containing adaptor-inducing IFN-*β* (TRIF)-dependent pathway in TLR signaling. *The Journal of Immunology*.

[45] Sato S., Sugiyama M., Yamamoto M. (2003). Toll/IL-1 receptor domain-containing adaptor inducing IFN-*β* (TRIF) associates with TNF receptor-associated factor 6 and TANK-binding kinase 1, and activates two distinct transcription factors, NF-*κ*B and IFN-regulatory factor-3, in the toll-like receptor signaling. *The Journal of Immunology*.

[46] Puccetti P., Grohmann U. (2007). IDO and regulatory T cells: a role for reverse signalling and non-canonical NF-*κ*B activation. *Nature Reviews Immunology*.

[47] Glenn J. D., Whartenby K. A. (2014). Mesenchymal stem cells: emerging mechanisms of immunomodulation and therapy. *World Journal of Stem Cells*.

[48] Prasanna S. J., Gopalakrishnan D., Shankar S. R., Vasandan A. B. (2010). Pro-inflammatory cytokines, IFN*γ* and TNF*α*, influence immune properties of human bone marrow and Wharton jelly mesenchymal stem cells differentially. *PLoS ONE*.

[49] Ren G., Zhao X., Zhang L. (2010). Inflammatory cytokine-induced intercellular adhesion molecule-1 and vascular cell adhesion molecule-1 in mesenchymal stem cells are critical for immunosuppression. *The Journal of Immunology*.

[50] Li X., Bai J., Ji X., Li R., Xuan Y., Wang Y. (2014). Comprehensive characterization of four different populations of human mesenchymal stem cells as regards their immune properties, proliferation and differentiation. *International Journal of Molecular Medicine*.

[51] Yang S. H., Park M., Yoon I. (2009). Soluble mediators from mesenchymal stem cells suppress T cell proliferation by inducing IL-10. *Experimental and Molecular Medicine*.

[52] Bassi E. J., Aita C. A. M., Câmara N. O. S. (2011). Immune regulatory properties of multipotent mesenchymal stromal cells: where do we stand?. *World Journal of Stem Cells*.

[53] Soleymaninejadian E., Pramanik K., Samadian E. (2012). Immunomodulatory properties of mesenchymal stem cells: cytokines and factors. *American Journal of Reproductive Immunology*.

[54] Di Nicola M., Carlo-Stella C., Magni M. (2002). Human bone marrow stromal cells suppress T-lymphocyte proliferation induced by cellular or nonspecific mitogenic stimuli. *Blood*.

[55] DelaRosa O., Lombardo E., Beraza A. (2009). Requirement of IFN-*γ*-mediated indoleamine 2,3-dioxygenase expression in the modulation of lymphocyte proliferation by human adipose-derived stem cells. *Tissue Engineering Part A*.

[56] English K., Barry F. P., Field-Corbett C. P., Mahon B. P. (2007). IFN-*γ* and TNF-*α* differentially regulate immunomodulation by murine mesenchymal stem cells. *Immunology Letters*.

[57] Kaisho T., Akira S. (2005). Toll-like receptors. *Encyclopedia of Life Sciences (eLS)*.

[58] Wang S., Li X., Zhao R. C. (2016). Transcriptome analysis of long noncoding RNAs in Toll-like receptor 3-activated mesenchymal stem cells. *Stem Cells International*.

[59] Chen D., Ma F., Xu S. (2013). Expression and role of Toll-like receptors on human umbilical cord mesenchymal stromal cells. *Cytotherapy*.

[60] Cho H. H., Bae Y. C., Jung J. S. (2006). Role of toll-like receptors on human adipose-derived stromal cells. *Stem Cells*.

[61] Raicevic G., Najar M., Stamatopoulos B. (2011). The source of human mesenchymal stromal cells influences their TLR profile as well as their functional properties. *Cellular Immunology*.

[62] Tomchuck S. L., Zwezdaryk K. J., Coffelt S. B., Waterman R. S., Danka E. S., Scandurro A. B. (2008). Toll-like receptors on human mesenchymal stem cells drive their migration and immunomodulating responses. *STEM CELLS*.

[63] Raicevic G., Rouas R., Najar M. (2010). Inflammation modifies the pattern and the function of Toll-like receptors expressed by human mesenchymal stromal cells. *Human Immunology*.

[64] Liotta F., Angeli R., Cosmi L. (2008). Toll-like receptors 3 and 4 are expressed by human bone marrow-derived mesenchymal stem cells and can inhibit their T-cell modulatory activity by impairing notch signaling. *Stem Cells*.

[65] Lombardo E., Delarosa O., Mancheño-Corvo P., Menta R., Ramírez C., Büscher D. (2009). Toll-like receptor-mediated signaling in human adipose-derived stem cells: implications for immunogenicity and immunosuppressive potential. *Tissue Engineering Part A*.

[66] Sangiorgi B., De Freitas H. T., Schiavinato J. L. (2016). DSP30 enhances the immunosuppressive properties of mesenchymal stromal cells and protects their suppressive potential from lipopolysaccharide effects: a potential role of adenosine. *Cytotherapy*.

[67] Lester S. N., Li K. (2014). Toll-like receptors in antiviral innate immunity. *Journal of Molecular Biology*.

[68] Romieu-Mourez R., François M., Boivin M.-N., Bouchentouf M., Spaner D. E., Galipeau J. (2009). Cytokine modulation of TLR expression and activation in mesenchymal stromal cells leads to a proinflammatory phenotype. *Journal of Immunology*.

[69] Park K. S., Kim S. H., Das A. (2016). TLR3-/4-priming differentially promotes Ca^2+^ signaling and cytokine expression and Ca^2+^-dependently augments cytokine release in hMSCs. *Scientific Reports*.

[70] Giuliani M., Bennaceur-Griscelli A., Nanbakhsh A. (2014). TLR ligands stimulation protects MSC from NK killing. *STEM CELLS*.

[71] Zhao X., Liu D., Gong W. (2014). The toll-like receptor 3 ligand, poly(I:C), improves immunosuppressive function and therapeutic effect of mesenchymal stem cells on sepsis via inhibiting MiR-143. *Stem Cells*.

[72] Fuenzalida P., Kurte M., Fernández-O'ryan C. (2016). Toll-like receptor 3 pre-conditioning increases the therapeutic efficacy of umbilical cord mesenchymal stromal cells in a dextran sulfate sodium–induced colitis model. *Cytotherapy*.

[73] Mancheño-Corvo P., Menta R., Del Río B. (2015). T lymphocyte prestimulation impairs in a time-dependent manner the capacity of adipose mesenchymal stem cells to inhibit proliferation: role of interferon *γ*, poly I:C, and tryptophan metabolism in restoring adipose mesenchymal stem cell inhibitory effect. *Stem Cells and Development*.

[74] Wang X., Cheng Q., Li L. (2012). Toll-like receptors 2 and 4 mediate the capacity of mesenchymal stromal cells to support the proliferation and differentiation of CD34^+^ cells. *Experimental Cell Research*.

[75] Cassatella M. A., Mosna F., Micheletti A. (2011). Toll-like receptor-3-activated human mesenchymal stromal cells significantly prolong the survival and function of neutrophils. *STEM CELLS*.

[76] Opitz C. A., Litzenburger U. M., Lutz C. (2009). Toll-like receptor engagement enhances the immunosuppressive properties of human bone marrow-derived mesenchymal stem cells by inducing indoleamine-2,3-dioxygenase-1 via interferon-*β* and protein kinase R. *Stem Cells*.

[77] Waterman R. S., Tomchuck S. L., Henkle S. L., Betancourt A. M. (2010). A new mesenchymal stem cell (MSC) paradigm: polarization into a pro-inflammatory MSC1 or an immunosuppressive MSC2 phenotype. *PLoS ONE*.

[78] Gray A., Maguire T., Schloss R., Yarmush M. L. (2015). Identification of IL-1*β* and LPS as optimal activators of monolayer and alginate-encapsulated mesenchymal stromal cell immunomodulation using design of experiments and statistical methods. *Biotechnology Progress*.

[79] Mei Y.-B., Zhou W.-Q., Zhang X.-Y., Wei X.-J., Feng Z.-C. (2013). Lipopolysaccharides shapes the human Wharton's jelly-derived mesenchymal stem cells in vitro. *Cellular Physiology and Biochemistry*.

[80] Krampera M., Cosmi L., Angeli R. (2006). Role for interferon-*γ* in the immunomodulatory activity of human bone marrow mesenchymal stem cells. *Stem Cells*.

[81] Tsan M. F., Gao B. (2004). Endogenous ligands of Toll-like receptors. *Journal of Leukocyte Biology*.

[82] Zhou Y., Guo M., Wang X. (2013). TLR3 activation efficiency by high or low molecular mass poly I:C. *Innate Immunity*.

[83] Krug A., Rothenfusser S., Hornung V. (2001). Identification of CpG oligonucleotide sequences with high induction of IFN-alpha/beta in plasmacytoid dendritic cells. *European Journal of Immunology*.

[84] Krieg A. M. (2002). CpG motifs in bacterial DNA and their immune effects. *Annual Review of Immunology*.

[85] van Ojik H. H., Bevaart L., Dahle C. E. (2003). CpG-A and B oligodeoxynucleotides enhance the efficacy of antibody therapy by activating different effector cell populations. *Cancer Research*.

[86] Vollmer J., Weeratna R., Payette P. (2004). Characterization of three CpG oligodeoxynucleotide classes with distinct immunostimulatory activities. *European Journal of Immunology*.

[87] Sivori S., Carlomagno S., Moretta L., Moretta A. (2006). Comparison of different CpG oligodeoxynucleotide classes for their capability to stimulate human NK cells. *European Journal of Immunology*.

[88] Jiang H., Milo R., Swoveland P., Johnson K. P., Panitch H., Dhib-Jalbut S. (1995). Interferon *β*-lb reduces Interferon *γ*-induced antigen-presenting capacity of human glial and B cells. *Journal of Neuroimmunology*.

[89] Ferrara J. L. M., Levine J. E., Reddy P., Holler E. (2009). Graft-versus-host disease. *The Lancet*.

[90] Guo B., Chang E. Y., Cheng G. (2008). The type I IFN induction pathway constrains Th17-mediated autoimmune inflammation in mice. *The Journal of Clinical Investigation*.

[91] Liu Y., Carlsson R., Comabella M. (2014). FoxA1 directs the lineage and immunosuppressive properties of a novel regulatory T cell population in EAE and MS. *Nature Medicine*.

[92] Compston A., Coles A. (2002). Multiple sclerosis. *The Lancet*.

[93] Reder A. T., Oger J. F., Kappos L., O'Connor P., Rametta M. (2014). Short-term and long-term safety and tolerability of interferon *β*-1b in multiple sclerosis. *Multiple Sclerosis and Related Disorders*.

[94] Amarnath S., Foley J. E., Farthing D. E. (2015). Bone marrow-derived mesenchymal stromal cells harness purinergenic signaling to tolerize human Th1 cells in vivo. *Stem Cells*.

